# Subcutaneous Injection of Myrrh Essential Oil in Mice: Acute and Subacute Toxicity Study

**DOI:** 10.1155/2019/8497980

**Published:** 2019-03-03

**Authors:** Ramakanta Lamichhane, Kyung-Hee Lee, Prakash Raj Pandeya, Kang-Kyung Sung, SangKwan Lee, Yun-Kyung Kim, Hyun-Ju Jung

**Affiliations:** ^1^Wonkwang University, College of Pharmacy, Department of Oriental Pharmacy & Wonkwang Oriental Medicines Research Institute, Iksan, Jeonbuk, Republic of Korea; ^2^Department of Internal Medicine and Neuroscience, Gwangju Oriental Medical Hospital and Jangheung Integrative Medical Hospital, Wonkwang University, Republic of Korea; ^3^Clinical Trial Center, Wonkwang University Gwangju Hospital, Gwangju, Republic of Korea

## Abstract

Myrrh essential oil (MEO) is widely used as remedies for the different human ailment in different parts of the world. The misuse of this natural product in higher doses may lead to fever, inflammation, and liver and kidney problems. In this study, we performed the acute and subacute toxicity analysis of MEO in mice model after subcutaneous injection and evaluated the safe dose to prevent the possible risk and side effects. Initially (first phase study) higher dose of MEO (20, 40, and 80 *μ*L) was injected, and later in the second phase study lower dose of MEO (1, 5, and 10 *μ*L) was injected for three days in each group of mice. Blood samples were taken for the investigation of hematological parameters and activity of various enzymes. The liver, kidney, spleen, lungs, and heart were excised for histological study. The body weight and skin abnormalities were also evaluated. In the first phase study, the mice showed granuloma formation at the site of injection. The liver showed dilated sinusoids and enlarged central vein. In the spleen the distinction between red and white pulp was lost. The kidney showed the degeneration of glomerulus. The enzyme activity and body weight were also decreased by the higher dose. The WBC count also increased nearly by twofold. Pruritus and self-trauma were also evident. Later in the second phase study, the skin abnormalities (granuloma) and damage in the structure of tissue (in liver, spleen, and kidney) were absent along with no change in enzyme levels, blood parameters, and body weight compared to the control. The MEO was toxic to liver, spleen, and kidney in the higher doses. The safe volume of MEO useful for various studies in mice was evaluated. The safe use of MEO should be assured, it should not be misused, being considered as a natural remedy, and there should be awareness of its toxicity and side effects.

## 1. Introduction 

Myrrh has been used as medicine for long time [1]. It is widely used as home medicine in different parts of African and Arabian countries [2]. It is obtained from different species of* Commiphora *[3, 4]. Many biological studies on myrrh extract and fractions (obtained from hydrodistillation) have been reported [3, 4]. It has been reported that the myrrh can be used in the treatment of ulcers,* Schistosoma*,* Fasciolopsis*, respiratory catarrh,* Furunculosis*, and diabetes [5, 6]. Myrrh was also found to promote permeability of medicine from epidermis to dermal capillaries and also enhance the wound healing process [7, 8]. It consists of various chemical constituents like cadinene, elemol, eugenol, cuminaldehyde, numerous furanosesquiterpenes including furanogermacranes, furanodiene, furanodienone, curzerene, and lindestrene, as well as furanoeudesma-1,3-diene [9, 10].

Myrrh essential oil (MEO) is prepared by the fractional distillation of the resins (myrrh) obtained from the plant* Commiphora myrrha* [11]. The volatile MEO is thick and pale yellow in colour. Along with various medicinal values of MEO, there are some toxicity and side effects associated with it. It has some hepatotoxicity if used in higher doses [12]. MEO has also been found affecting kidney and heart while using in improper doses [13]. Since it is used topically most of the time, there may be many chances of hazards if precautions are not applied. In different research studies it has been used subcutaneously in animal models. Its various applications have been mentioned but its toxicity study has not been clearly and accurately reported. So, we considered it as an important aspect of research. This study is related to the acute and subacute toxicity of myrrh essential oil (MEO) and the hydrosol obtained during the preparation of the essential oil in the distillation plant. Due to its strong smell and unpleasant taste its oral administration is difficult. Making emulsion in water or other suitable medium would help to mask its bitter and disagreeable taste and improve patience acceptance and compliance [14]. Another method for MEO administration would be subcutaneous injection, like many other essential oils for various clinical studies [15, 16]. However subcutaneous injection of oil like materials can sometime be problematic if proper attention and procedures are not followed. Since the oil cannot diffuse easily into the tissue, it will not disperse completely and remains as a cyst [17]. Subcutaneous injection of oily substance (mineral, vegetable, or animal oil) may lead to the body reaction called oleoma or granulomatous reaction in the subcutaneous fatty tissue [18–20].

## 2. Materials and Methods

### 2.1. Myrrh Essential Oil and Hydrosol

The resin of plants* Commiphora myrrha* was obtained from My Ryeung Herbal Medicine, Co. Ltd., Seoul, Korea. The resin was grounded to power with the help of grinder. Then the steam distillation of the grounded resin was done in Clevenger apparatus, and the oil layer above the hydrosol layer was collected. Since MEO is partly soluble in water, the hydrosol layer below the oil layer was also collected as it also contained very small part of MEO [21]. The hydrosol also had weak typical odor of MEO. The toxicity of MEO and the hydrosol obtained during the preparation of MEO in the distillation plant were studied for the acute and subacute toxicity. Like MEO, there may be high chances of using the hydrosol for medicinal values or other purposes. So, we included the toxicity study of hydrosol also.

### 2.2. Animals

ICR mice (average weight 30 g) were taken for the animal experiment. All the mice were housed in mouse room maintaining standard environmental conditions of temperature (25±1°C), relative humidity (55±5%), and 12/12 h light dark cycle. They had free access to standard pellet diet and water* ad libitum.* After the adaptation period of four days, the experiment was started. All animal experiments were carried out in accordance with the guidelines for the care and use of laboratory animals by Wonkwang University.

### 2.3. First Phase Study

**Figure 1:**
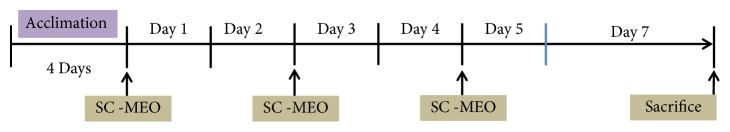
Experiment schedule for sample injection (SC^*∗*^ : subcutaneous injection). The different concentrations of MEO samples as mentioned in Table 1 were injected subcutaneously for three times.

Mice were randomly divided into five different groups (6 mice each) as below on the basis of sample injected subcutaneously (Table 1). Control was injected with normal saline. F-200 group was injected with hydrosol obtained during distillation of MEO. The other groups were injected with MEO in different volumes as indicated in Table 1. The subcutaneous injection was given over the shoulders, into the loose skin over the neck [22]. MEO samples were prepared by mixing the oil and normal saline with final volume 100 *μ*L and shaking vigorously before injection. All samples were injected three times as shown in experiment schedule, Figure 1. The mice were sacrificed on the 7th day.

### 2.4. Second Phase Experiment

Since we observed some level of toxicity in first study, we did second phase study with lower dose of MEO. Mice were randomly divided into four groups (6 mice each) as shown in Table 2. After the adaptation period, oil samples were prepared by mixing with normal saline vigorously right before the injection (subcutaneous) in the ratios shown in Table 2. The schedule of injection of sample is explained in Figure 1. The control groups were injected with normal saline subcutaneously. The mice were sacrificed on the 7^th^ day.

### 2.5. Observations

The mice were observed daily to check if there was any mortality or morbidity. Any types of swelling on the skin at or near the site of injection were also examined throughout the experiment period. The body weight and food intake of each group of mice were measured daily. The mice were checked daily for any skin abnormalities or wound due to self-scratching.

### 2.6. Clinical Parameters and Biological Assay after Sacrifice

Investigation of hematological parameters is very essential in toxicity evaluation as they help to determine the extent of deleterious effect of foreign compound on the blood [23, 24]. Blood samples for the hematology were obtained from the eye of the mice from the orbital sinus [25]. EDTA was used as an anticoagulant for hematology samples. The hematology variables (Table 3) were evaluated in Genius Auto Hematology Analyzer (Model: KT-6200 Vet). After the hematological assay the blood sample was centrifuged (15000 rpm, 15 min, 4°C) and plasma was separated for enzyme assay. Aspartate aminotransferase (AST) and alanine aminotransferase (ALT) levels in plasma were measured by using a commercially available kit (AM101-K; Asan Pharm., Korea). Renal function test was performed by evaluating blood urea nitrogen (BUN) and creatinine (CRE) level, using diagnostic kits “AM 165-K, Asan Pharm Co., Ltd, Hwaseong, Korea” and “AM 119-K, Asan Pharm Co., Ltd, Hwaseong, Korea”, respectively. All experiments were assayed by manufacturer's protocol, and reaction solutions were analyzed using UV-vis spectrophotometer.

### 2.7. Histology

After the sacrifice, the liver, kidney, spleen, lungs, and heart were excised from dissected mice, washed with PBS (phosphate buffer saline), and weighed. Then they were fixed in 10% formalin for histopathological examination. The organ or tissues were processed, embedded in paraffin, sliced, and stained with hematoxylin and eosin according to the standard technique [26]. The sections were carefully examined under the microscope. The histopathological changes deviant from the normal were carefully recorded.

### 2.8. Statistical Analysis

The results are expressed as a mean ± standard deviation (S.D.). Blood biochemical parameters and liver enzymes were analyzed by one-way analysis of variance (ANOVA) using IBM SPSS Statistics version 22.0 (IBM Corp., Armonk, NY, USA) and the body weight change was analyzed by t-test using Microsoft Excel 2010 software. For all analyses, p<0.05 was considered statistically significant.

## 3. Results 

### 3.1. First Phase Experiment

#### 3.1.1. Mice Behavior, Skin Abnormalities, and Injury 

**Figure 2:**
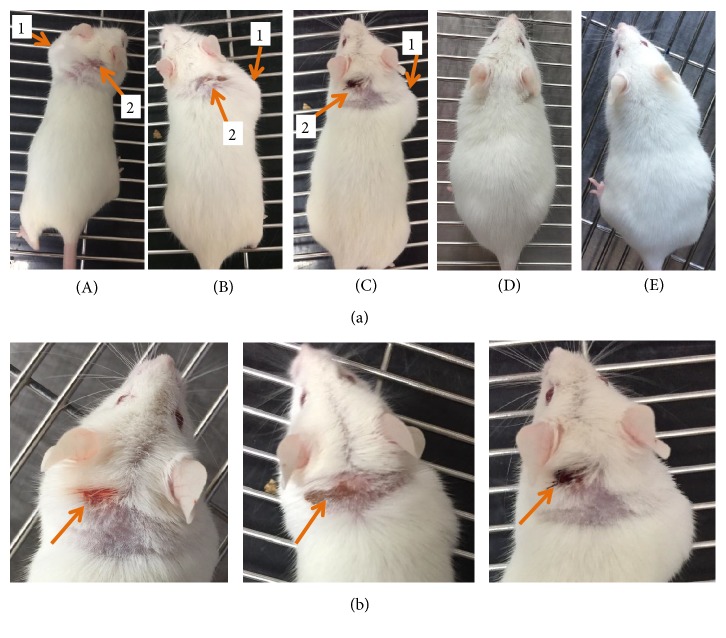
Observation of physiological changes in skin after subcutaneous injection of MEO. (a) Mice were injected with sample containing 20 *μ*L (A), 40 *μ*L (B), and 80 *μ*L (C) of MEO and 200 *μ*L of hydrosol formulation (D) and normal saline (E), and the pictures were taken at the last day of experiment before sacrifice to evaluate any skin abnormalities. The MEO treated groups (A, B, and C) showed swelling (1) and some sort of wound (2) due to scratches by themselves. The F-200 (D) and normal saline (E) group did not show any kind of skin abnormalities. (b) Significant abrasions on the ears and neck region of mice in MEO (20, 40, and 80) groups.

Mice were observed every day throughout the experiment. The injection was done subcutaneously into the loose skin over the neck (dorsal part). Skin abnormalities, as shown in Figure 2(a), were seen in all mice belonging to MEO (20, 40, and 80) groups from the next day of subcutaneous injection. Palpable subcutaneous nodule (at a little distance far from the site of injection) was seen on the right or left shoulder-neck region of the mice. In addition, pruritus (unpleasant sensation that provokes the desire to scratch) and self-trauma were also evident as they had scratches at the subscapular region (dorsal surface of neck) proximal to the site of injection leading to abrasions (Figure 2(b)). The control and F-200 group mice did not show any such type of skin abnormalities (subcutaneous nodule, scabbing, or abrasions). The swollen subcutaneous nodule enlarged slowly and became filled with pus like material by the time of sacrifice. The excision of nodule at the time of sacrifice revealed that it contained some parts of MEO trapped within (from the odor). The lipogranuloma nodule is formed generally as a result of aggregation of macrophage after the entry of degradation-resistant lipid deposits in the subcutaneous region [20]. Since there was no death till the last day of experiment, the Lethal dose (LD_50_) for the MEO can be more than 80 *μ*L.

#### 3.1.2. Effect on Blood Clinical Parameters

**Figure 3:**
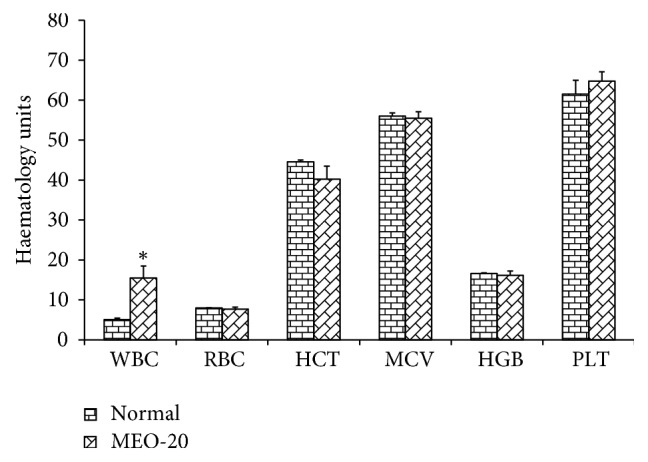
Hematological parameters analysis for MEO 20 and control groups. Values are expressed as mean ± standard deviation (n = 6). Statistical significance was calculated using one-way ANOVA followed by Dunnett's multiple comparison test. ^*∗*^P < 0.05 vs control.

Different hematological parameters of the blood samples of mice were measured, and the results are shown in the bar diagram (Figure 3). In MEO (20, 40, and 80) injected groups, only the WBC count increased around double compared to the control group, while there was no significant deviation in other hematological parameters compared to the control (data shown for only the samples of MEO 20). In F-200 the hematological parameters were similar to that of control (data not shown) indicating no abnormalities in the hematological parameters. It has been suggested that the increase in WBC count is mainly associated with acute infection, inflammation, or tissue damage [27]. So, the increased WBC in MEO (20, 40, and 80) group is associated with the lipogranuloma at the shoulder-neck region.

#### 3.1.3. Effect on Liver and Kidney (Biochemical Assay)

For the evaluation of liver and kidney toxicity due to oil injections, the activity of liver and kidney enzymes was measured and the results are shown in Table 4. The serum AST level significantly increased in MEO 20, 40, and 80 groups compared to the control indicating toxicity in liver. However, the serum level of ALT was found to decrease with increase in volume of MEO injection in comparison to the control. The higher level of CRE in MEO 20, 40, and 80 compared to control indicated the kidney toxicity. However, the BUN level was found to decrease in MEO 20, 40, and 80 compared to control. The serum AST, ALT, CRE, and BUN values for F-200 were similar to that of control (Table 4), indicating no toxicity to liver and kidney.

#### 3.1.4. Food Intake and Body Weight

**Figure 4:**
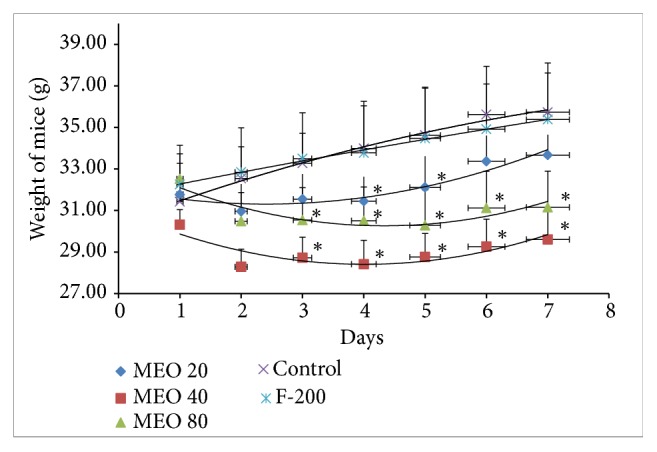
Body weight change in mice during the experiment period. Values are expressed as mean ± standard deviation (n = 6). Statistical significance was calculated using t-test. ^*∗*^P < 0.05 vs control.

**Figure 5:**
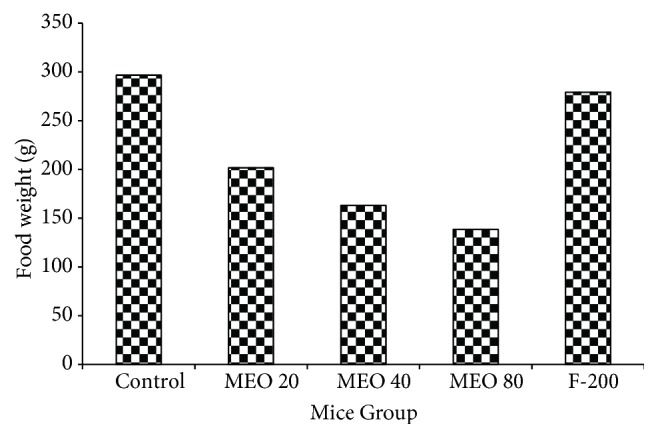
Total food intake pattern of each group of mice during the seven-day experiment. Values are expressed as mean ± standard deviation (n = 6).

The body weight and food intake amount gradually decreased after the subcutaneous injection of MEO (20, 40, and 80) as shown in Figures 4 and 5, respectively. MEO 40 and 80 showed sharp decrease in body weight till day 4, and after that there was slight gain in body weight, but the initial body weight could not be returned back. MEO 20 initially lost the body weight but later slowly gained body weight a little more than the initial body weight. The control and F-200 groups showed significant increase in body weight. The food intake pattern also supported the results of body weight change. There was sharp decrease in food intake after MEO injection. The MEO 80 showed the lowest quantity food intake followed by MEO 40 and MEO 20. The food intake amount decreased with the increase in volume of MEO injection. The F-200 group showed similar pattern of food intake like control. The decrease in food intake might be due to the pain and discomfort due to inflammation and granuloma after oil injection.

#### 3.1.5. Organ Weight

**Figure 6:**
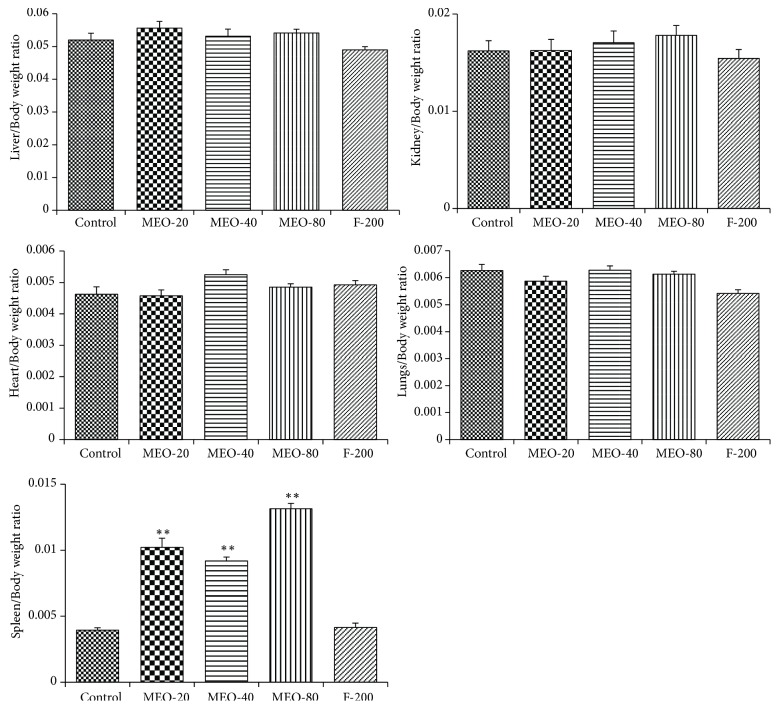
Change in weight of different organs after subcutaneous injection of MEO. Values are expressed as mean ± standard deviation (n = 6). Statistical significance was calculated using one-way ANOVA followed by Dunnett's multiple comparison test. ^*∗∗*^P < 0.01 vs control.

Toxicity of drugs, chemicals, or any foreign material can also be evaluated from the change in the physiological structure and weight of the organ. The data in Figure 6 show the results of the weight of different organs of all experimental groups of mice. Except spleen, there has been no significant change in weight of organ compared to that of control. All of the MEO (20, 40, and 80) groups showed increase in size and weight of spleen compared to the control. Increase in spleen is mainly attributed to the systemic inflammation. So, even the lowest volume of 20 *μ*L was also capable of inducing greater systemic inflammation and leading to splenomegaly. The formulation F-200 had no such negative effect on spleen and other organs.

#### 3.1.6. Histology

**Figure 7:**
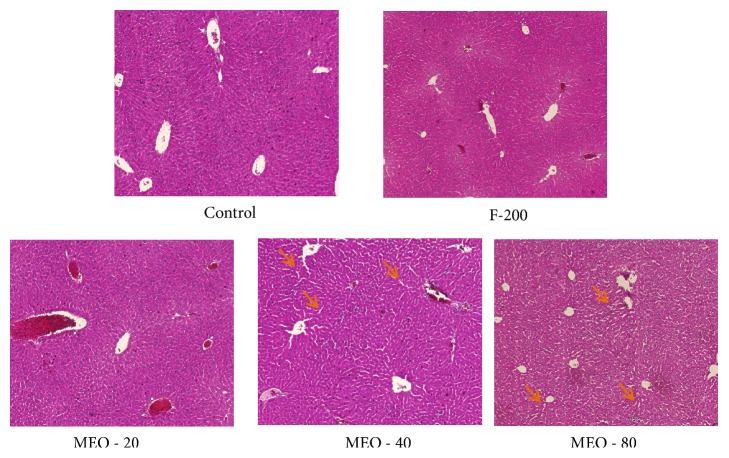
Histology of liver after hematoxylin and eosin staining (× 40 magnification). Small arrow (orange colour) indicates the swelling sinusoids.

**Figure 8:**
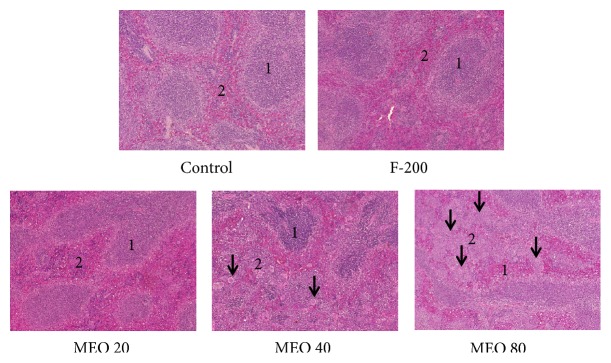
Histology of spleen after hematoxylin and eosin staining (× 40 magnification). Control and F-200 showed distinct spleen follicle with clear white pulp (1), red pulp (2), and marginal zone. White pulp and red pulp are more irregular with increase in dose of MEO. Accumulation of megakaryocytes (small arrow) was found in huge number in MEO 40 and 80.

**Figure 9:**
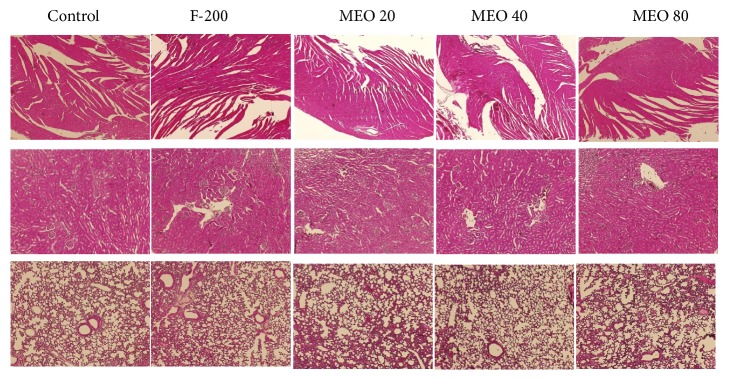
Histology of kidney, lungs, and heart of different groups of mice after hematoxylin and eosin staining (× 40 magnification). MEO 80 showed the congestion of glomerulus compared to control. No abnormalities in the structure of heart and lungs are seen in the histology of MEO 20, 40, and 80 mice.

The histopathological investigations of liver, kidney, spleen, heart, and lungs were done to find out any morphological changes indicating toxicity or damage to the tissue. The histological section of liver Figure 7 revealed the presence of dilated sinusoids and central vein in MEO 20, 40, and 80. The damage was severe in MEO 80 followed by MEO 40. MEO 20 had caused less damage to the liver. In the spleen and kidney also there was disorder due to injection of MEO. The histology of spleen in Figure 8 revealed general disorganization, apparent loss of distinction between the red and white pulps, and extensive accumulation of megakaryocytes. The MEO 80 group had the greatest degree of damage in normal structure of spleen and highest accumulation of megakaryocytes. The kidney showed the presence of disorganization in tubules and degeneration of glomerulus mainly in MEO 80. MEO 40 and 20 showed very little effect on kidney (Figure 9). This suggests that higher dose of MEO was toxic to liver, kidney, and spleen. The microanatomy of liver, kidneys, and spleen did not present any treatment-related adverse effects in the control and F-200 group (Figures 7, 8, and 9). The lungs and heart histological study did not show any kind of abnormalities in all groups as investigated from Figure 9.

### 3.2. Second Phase Experiment

#### 3.2.1. Mice Behavior, Skin Abnormalities, and Injury

In the first phase experiment we found that even the lowest dose of 20 *μ*L injection of MEO caused skin abnormalities and toxicity in certain organs in mice. So in the second experiment we evaluated the effects of lower doses of MEO (1, 5, and 10 *μ*L). We mixed the oil with water (final volume 100 *μ*L) and shook vigorously before injection in mice.

**Figure 10:**
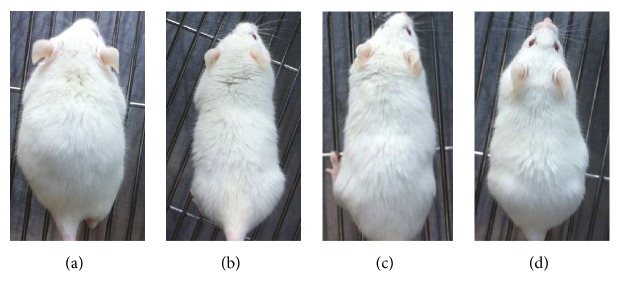
Mice were injected with sample containing 1 *μ*L (a), 5 *μ*L (b), 10 *μ*L (c), and normal saline (d), and the pictures were taken at the last day of experiment before sacrifice to evaluate any skin abnormalities. The sample treated groups showed no such abnormalities (swelling and wound) seen in higher doses. The sample groups had similar skin condition like that of normal saline treated groups.

None of the groups (MEO 1, 5, and 10) showed any kind of skin swelling or abnormalities after the MEO injection, which can be confirmed from Figure 10. There was no scratch around the injection area of the body. In addition to that, MEO 1, 5, and 10 had no such skin problems after the injection (Figure 10).

#### 3.2.2. Effect on Blood Clinical Parameters

**Figure 11:**
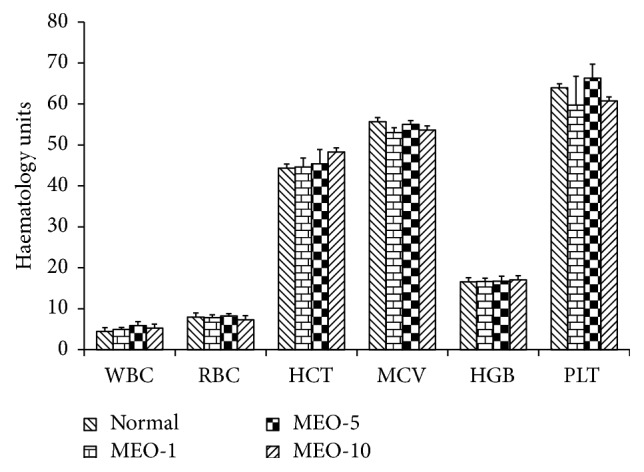
Hematological parameters analysis for MEO (1, 5, and 10) and control groups. Values are expressed as mean ± standard deviation (n = 6). Statistical significance was calculated using one-way ANOVA followed by Dunnett's multiple comparison test.

The different blood parameters of blood samples of MEO (1, 5, and 10) and control were evaluated and the results are shown in Figure 11. The MEO group and control showed similar data for the hematological parameters of the blood samples. This indicated that the subcutaneous injection of MEO up to 10 *μ*L was safe and did not induce toxic effects in blood.

#### 3.2.3. Effect on Liver and Kidney (Biochemical Assay)

The results of measurement of activity of liver and kidney enzymes are shown in Table 5. The results indicated that there was no significant change in the activity of enzymes after the administration of MEO (1, 5, and 10) as compared to that of control. This suggested that there was no toxic effect in the liver and kidney due to subcutaneous injection of MEO (1, 5, and 10).

#### 3.2.4. Food Intake and Body Weight

**Figure 12:**
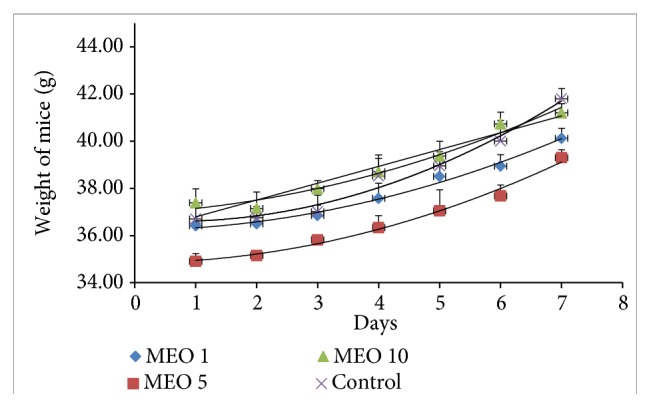
Body weight change in mice during the experiment period. Values are expressed as mean ± standard deviation (n = 6) for the measurement on each day. Statistical significance was calculated using t-test.

**Figure 13:**
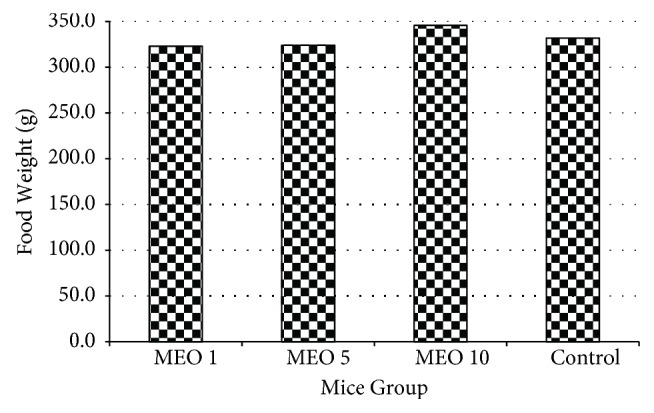
Total food intake pattern of each group of mice during the seven-day experiment. Values are expressed as mean ± standard deviation (n = 6).

The changes in body weight and total food intake of each group throughout the experiment are given in Figures 12 and 13, respectively. The body weight of all groups (MEO 1, 5, and 10) increased right from the beginning after the injection. The body weight gain in MEO 1, 5, and 10 was similar to that of control at the end of the experiment. The total food intakes of the sample and control groups were similar (Figure 13). It suggested that MEO 1, 5, and 10 had no such severe effects which would hamper the appetite of mice.

#### 3.2.5. Organ Weight

**Figure 14:**
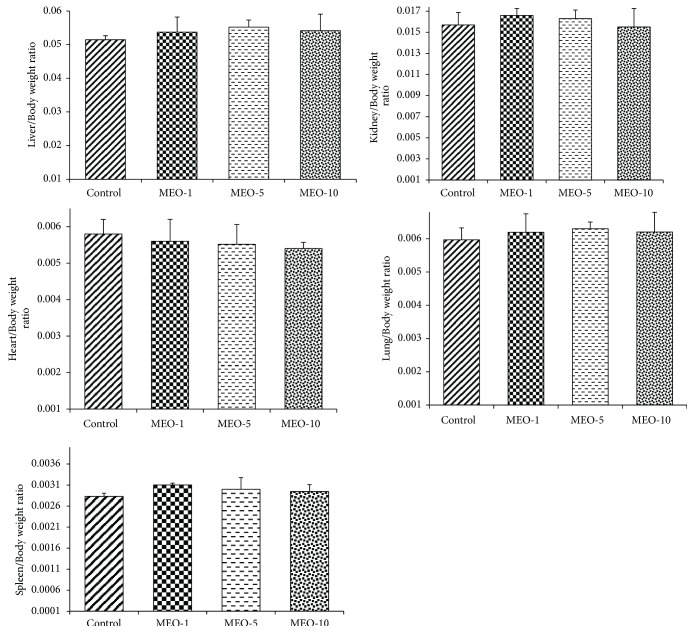
Change in weight of different organs after subcutaneous injection of MEO. Values are expressed as mean ± standard deviation (n = 6). Statistical significance was calculated using one-way ANOVA followed by Dunnett's multiple comparison test.

The data in Figure 14 show the results of the weight of different organs of all experimental groups of mice of second phase study. There was no such significant difference in organ weight of MEO (1, 5, and 10) and control. It suggests that the MEO (1, 5, and 10) had no such adverse effects on liver, kidney, spleen, hart, and lungs.

#### 3.2.6. Histology

**Figure 15:**
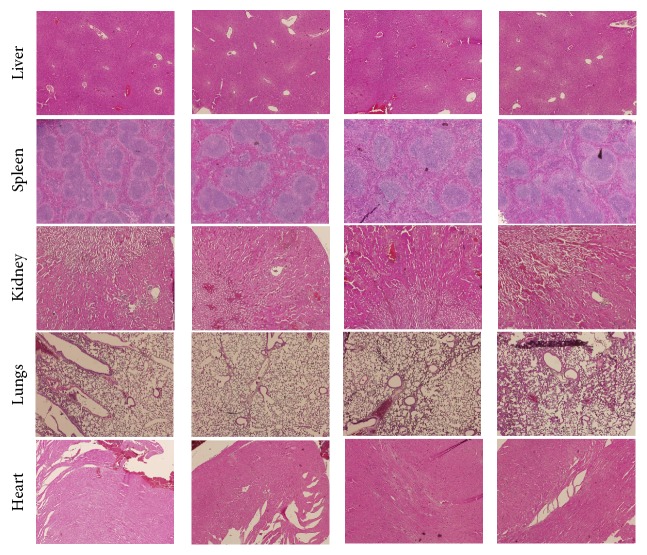
Histology of organs of mice in the second phase experiment after hematoxylin and eosin staining (× 40 magnification). No abnormalities in the structure of liver, spleen, kidney, lungs, and heart are seen in the histology of organs of mice injected with MEO 1, 5, 10 and normal saline (control).

Like in the first phase study, the mice of second phase study were sacrificed, and the five organs, namely, liver, kidney, spleen, heart, and lungs, were taken for the histopathological study. The histological sections of all organs are shown in Figure 15 and reveal that there were no such pathological abnormalities in the tissues. They looked healthy as those of control groups.

## 4. Discussion 

The use of natural products for treatment of different human ailments is increasing day by day. Toxicity evaluation of such natural products is very important for their safe and correct use. Sometimes the natural products may not be toxic but its higher dose may induce the toxicity. Sometimes they may be toxic to a particular organ in the body causing some tissue damage or metabolic dysfunction. So, from the toxicity evaluation, we get much useful information about the natural products for their safety and effective use. Likewise, this study was designed to evaluate the toxicity of myrrh essential oil (MEO), an essential oil with various medicinal values. Its use is increasing day by day in different countries. Its various biological activities have been reported, but its toxicity and safe evaluation have not been studied in detail yet. In this study we tried to establish the toxicity of MEO by injecting it subcutaneously in various doses to mice.

The main chemical components of MEO are *α*-pinene, cadinene, limonene, cuminaldehyde, eugenol, m-cresol, heerabolene, acetic acid, formic acid, and other monoterpenes, sesquiterpenes, alcohols, and esters [28]. They are responsible for various biological activities and unique odor to MEO.

Acute and subacute toxicity of subcutaneous injection of MEO was studied in mice model. In the first phase of study the injection of higher doses of MEO (20, 40, and 80) caused the formation of nodule below the skin surface after 24 hours. It has been reported that the entry of foreign body which does not disperse quickly in skin leads to body reaction which induces accumulation of macrophage, lymphocytes, and fibroblasts to the site of injections [29, 30]. Hence, the nodule was induced by body reaction or inflammation due to the slow dispersion and degradation of MEO in the tissue. The nodule was incised at the time of sacrifice which was filled with pus and little amount of MEO (identified on the basis of its unique smell). Pruritus and self-trauma were also visible as the oil-injected mice were observed to scratch the subscapular region proximal to the site of injection. Some of them have also developed wound on the area near the site of injection. The study of blood parameters revealed the twofold increase in WBC counts in MEO (20, 40, and 80) groups compared to the control, confirming the inflammation along with the granuloma. The increase in WBC indicates that the immune system is working to destroy an infection [31]. However, it is not necessary that there is rise in WBC count due to granuloma. In a study the animals did not show any change in WBC accompanied with the granuloma [32]. In our study the inflammation must be present along with granuloma, which resulted in increase in WBC count. The granuloma will last until the oil is slowly eliminated from the reservoir. It may turn to big wound if any infection occurs in the granuloma. Sometimes if the granuloma is formed due to injection of nonbiodegradable materials, surgical removal of the injected material will be the only option of treatment [33].

Some studies have reported that the volume of oil also determines the formation of granuloma [34]. In our second phase study, the injection of MEO oil in lower doses (1, 5, and 10 *μ*L) surprisingly escaped the occurring of granuloma in the mice. Not only that, in the MEO (1, 5 and 10) groups we did not observe skin inflammation, swelling, dermatitis, scabbing, and abrasions along the neck, shoulders, and/or ears near the site of injection. The blood parameters were also found in normal range compared to the control in the MEO (1, 5, and 10) groups. The lower volumes of MEO up to 10 *μ*L would be effective in preventing the skin inflammation or granuloma formation. The important thing is that the final volume of injection was 100 *μ*L after mixing with normal saline. While mixing, the smaller volumes of MEO (1, 5, and 10 *μ*L) formed good suspension of fine particles in the normal saline. While the large volumes of MEO (20, 40, and 80 *μ*L) did not show such good suspensions after mixing with normal saline, and clear separation of oil and aqueous phase was visible. So, the large volume of oil might have played role in formation of glomerulus. Hence, this study has been able to find safe injection volume for MEO in mice to avoid granuloma and trauma in the subcutaneous injection study.

Different studies have shown that an elevation in the activity of these liver and kidney enzymes (ALT, AST, CRE, and BUN) is conventionally an indicator of liver and kidney injury [35, 36]. So, ALT, AST, CRE, and BUN are important markers for the detection of damage in liver and kidney. The injection of MEO (20, 40, and 80) significantly influenced the activity of ALT, AST, CRE, and BUN compared to the control. The serum AST level significantly increased in MEO 20, 40, and 80 groups compared to the control. This indicates the toxicity of MEO right from its lowest dose of 20 *μ*L. The serum level of ALT was found to decrease with increase in volume of MEO injection in comparison to the control. According to some research findings this situation has been linked with the consequence of death of liver cells and decrease in the amount of production of such liver markers [37]. After all, the increase in AST level due to injection of MEO 20, 40, and 80 strongly proves that there is toxicity. Liver enzymes (AST, ALT) are present in maximum amount in liver cells. So, any damage to these cells due to necrosis or increase permeability of cell membrane may increase the leakage of these enzymes and their levels sharply increase in the blood [38]. So, in case MEO induces toxicity, there might be sinusoidal dilation induced necrosis leading to apoptosis of liver cells and increase in liver enzymes in the blood.

The increase in level of serum CRE in MEO 20, 40, and 80 compared to control indicates kidney toxicity. This is mainly associated with the decrease in glomerular filtration rate of kidney due to some damage in the glomerulus. However the level of BUN which was found to decrease in MEO 20, 40, and 80 groups could be the subject matter of further study to find whether it happened due to toxicity or not. Overall, the increase in prominent liver and kidney enzymes proves that the injection volume of MEO 20, 40, and 80 would be toxic to liver and kidney in mice. Further study was done on lower volumes of MEO (1, 5, and 10 *μ*L) to study the effect on liver and kidney. The levels of all enzymes (ALT, AST, CRE, and BUN) were found in normal range in MEO (1, 5, and 10 *μ*L) groups compared to control. Hence, decreasing the injection volume to 1, 5, and 10 *μ*L could be a safe volume for MEO in mice.

In the histological study, the administration of MEO (20, 40, and 80) caused changes in normal structure of liver and spleen in majority. The MEO 40 and 80 greatly dilated the sinusoids (liver) followed by MEO 20. This sort of toxic effect in liver has been observed in other toxic studies also [39]. All the MEO 20, 40, and 80 groups showed splenomegaly. With the increase in dose (MEO 20, 40, and 80) there was greater infiltration of macrophages (histology study) in the spleen. The histology showed that there was no distinction between red pulp and white pulp in the spleen due to depopulation of the white pulp hypercellularity. The sharp increase in WBC in MEO (20, 40, and 80) groups compared to control further confirms the skin inflammation and spleen toxicity. Similar type of toxic effect (splenomegaly and sharp increase in WBC) in spleen was also recorded in a study of subcutaneous injection of higher dose of calcium carbonate nanoparticles [38]. In the lower dose MEO (1, 5, and 10 *μ*L) groups, the histology of liver, spleen, and kidney revealed no such abnormalities in the structure compared to the control. From the histology of other organs (heart and lungs), it was surprising that even the higher volumes (40 and 80 *μ*L of MEO) did not affect the normal structure.

The overall study has reflected the toxicity of MEO in higher doses and explained how the organs are affected by the toxicity. So, strict precaution should be applied while using MEO along with other medications which have similar toxicity to MEO. Otherwise the toxic effect would be intensified and may impose severe impact on the function of organs. This study has also been able to extend the knowledge of the toxic effect of natural products which may help people to be more conscious while using natural products. Further study in human should be done to evaluate the actual dose leading to toxicity in human which may vary from the animal study. Another limitation was that without further studies it is hard to find to what level we can correlate the results of this study to human. The major components of MEO are reported to be furanoeudesma-1,3-diene (34 %), furanodiene (19.7 %), and lindestrene (12.0 %) [12]. These components may have some or no role in the toxicity to the organs in higher doses. This was also a limitation of this study as we could not figure out its major toxic component.

## 5. Conclusion

The overall results showed that the lower doses of MEO 1, 5, and 10 are safe as they had no skin nodule/inflammation and physiological damage to the organs. The higher doses of MEO 20, 40, and 80 were toxic causing damage to some organs (liver, kidney, and spleen). This study will also help to evaluate the doses of MEO in various preclinical studies (in rodents) which utilize MEO directly as a drug or indirectly as a vehicle for solubilizing the lipophilic drugs.

## Figures and Tables

**Table 1:** Experimental groups in first phase study along with the dose of samples injected subcutaneously for the toxicity study. The mixture of MEO and normal saline was made vigorously before injection.

Groups	Sample (volume)
1. Control	Normal saline (100 *μ*L)

2. MEO 80	80 *μ*L MEO + 20 *μ*L normal saline

3. MEO 40	40 *μ*L MEO + 60 *μ*L normal saline

4. MEO 20	20 *μ*L MEO + 80 *μ*L normal saline

5. F-200	Formulation of hydrosol (200 *μ*L)

**Table 2:** Experimental groups in second phase study along with the dose of samples injected subcutaneously for the toxicity study. The mixture of MEO and normal saline was made vigorously before injection.

Group	Sample
1. Control	100 *μ*L Normal saline

2. MEO 1	1 *μ*L MEO + 99 *μ*L normal saline

3. MEO 5	5 *μ*L MEO + 95 *μ*L normal saline

4. MEO 10	10 *μ*L MEO + 90 *μ*L normal saline

**Table 3:** List of hematological assay parameters measured after withdrawing the blood samples from the eye of the mice.

Hematological parameters	Units	Measurements
Red blood cell (RBC)	RBCx10^12^/L	Number of RBC counts

Hemoglobin (HGB)	g/dL	Hemoglobin concentration

Hematocrit (HTC)	%	Volume percentage (%) of red blood cells in blood

Mean Corpuscular Volume (MCV)	fL	Average size of red blood cells

White blood cells (WBC)	WBCx10^9^ /L	Number of WBC counts

Platelets (PLT)	PLTx10^9^/L	Number of Platelets counts

**Table 4:** Effect on the activity of liver and kidney enzymes. The blood samples of different group of mice were checked for the liver and kidney enzymes to evaluate the liver and kidney toxicity due to MEO injection in different doses. For the liver toxicity, the levels of AST and ALT enzymes were analyzed. Similarly for the kidney toxicity, the levels of BUN and CRE were analyzed.

Group	AST (U/L)	ALT (U/L)	BUN (U/L)	CRE (U/L)
Control	131.2±3.3	34.0±1.3	21.0±2.1	0.05±0.02

MEO 20	141.7±4.2^*∗*^	27.3±2.1^*∗*^	16.7±1.7^*∗*^	0.14±0.03^*∗*^

MEO 40	140.0±3.7^*∗*^	28.5±1.7^*∗*^	15.4±2.5^*∗*^	0.19±0.02^*∗*^

MEO 80	177.5±4.1^*∗*^	21.3±2.3^*∗*^	11.0±1.8^*∗*^	0.18±0.07^*∗*^

F-200	129.5±2.0	32.5±3.2	20.3±2.0	0.07±0.01

Values are expressed as mean ± standard deviation (n = 6). Statistical significance was calculated using one-way ANOVA followed by Dunnett's multiple comparisons test. ^*∗*^P < 0.05 vs control.

**Table 5:** Effect on activity of serum liver and kidney enzymes. The blood samples of different group of mice were checked for the liver and kidney enzymes to evaluate the liver and kidney toxicity due to MEO injection in different doses. For the liver toxicity, the levels of AST and ALT enzymes were analyzed. Similarly for the kidney toxicity, the levels of BUN and CRE were analyzed.

Group	AST(U/L)	ALT(U/L)	CRE(U/L)	BUN(U/L)
Control	156.44±3.1	39.11±1.4	0.053±0.03	22.81±1.7
MEO 1	147.71±2.3	36.92±2.3	0.045±0.10	19.52±1.3
MEO 5	150.82±4.3	37.71±3.1	0.061±0.01	23.12±2.1
MEO 10	148.92±2.6	37.23±2.8	0.059±0.06	20.91±1.9

Values are expressed as mean ± standard deviation (n = 6). Statistical significance was calculated using one-way ANOVA followed by Dunnett's multiple comparisons test.

## Data Availability

All the data are included in the manuscript. Any queries regarding the data would be considered upon request to the corresponding author through email.
